# Ritual Revision During a Crisis: The Case of Indian Religious Rituals During the COVID-19 Pandemic

**DOI:** 10.1177/07439156221081485

**Published:** 2022-07

**Authors:** Vikram Kapoor, Russell Belk, Christina Goulding

**Keywords:** religion, rituals, festivals, traditions, COVID-19, India

## Abstract

Rituals, particularly religious rituals, may play a significant role in times of crises. Often, these rituals undergo revision to adapt to the changing needs of the time. This article investigates recent unofficially revised Hindu religious rituals as performed during the COVID-19 pandemic. The multifarious creative interplay between Hindu tradition and change is illustrated through four cases: the religious festival of *Durga Puja*, the devotional songs or *bhajans*, the ritual of lighting lamps or *diyas*, and the fire rituals or *havans*. The authors offer a systematic discourse analysis of online news articles and YouTube posts that illuminate several aspects of ritual revision during unsettled times. They focus on the changes that were made to ritual elements: who controlled these alterations, how these modifications were made, and what potential benefits these revisions offered to the community of ritual participants. The authors highlight public policy implications regarding the involvement of diverse social actors, the creation of faith in science, the creation of feelings of unity and agency, and the amplification of local ritual modifications on a national scale.

Scholars of society and culture have long been interested in the study of rituals, their practices, and their meanings. A ritual, in general, is a symbolic activity performed in a specific order and repeated over time with fervor and sincerity (Rook 1985). Because of the widespread prevalence of rituals in modern cultures, they have been studied extensively from various perspectives. In marketing and consumer research, scholars have examined grooming rituals (Rook and Levy 1983), death rituals (Bonsu and Belk 2003), wedding rituals (Otnes and Scott 1996), gift-giving rituals (Minowa, Khomenko, and Belk 2011), rituals around Thanksgiving Day (Wallendorf and Arnould 1991), and more recently, interaction ritual chains (Hill, Canniford, and Eckhardt 2021). While these studies have contributed significantly to our understanding of rituals, there has been little reflection on ritual revision (cf. Bradford and Sherry 2013; Chitakunye and Maclaran 2014; Cross, Harrison, and Gilly 2017), particularly during unsettled times. The COVID-19 pandemic provided an opportunity for us to study ritual revision during a turbulent time. The idea of ritual revision, which was necessitated by the government's stay-at-home and social distancing policies, is central to this article. As such, we focus on public rituals at the intersection of consumer-initiated action, media and digital amplification, and public policy or political intervention.

During unsettled times, culture affects human behavior and actions in unexpected ways, causing people to reflect on themselves and their ideologies (Weinberger and Wallendorf 2012). In other words, situational investigations of consumer behaviors are essential, as some forms of behaviors “may be enacted only under specific circumstances” (Belk 1975, p. 157). The COVID-19 pandemic is an example of an unsettling period that has undermined our sense of life's stability and consistency (Campbell et al. 2020). Under these circumstances, many rituals, such as holidays, graduations, and weddings, have been redesigned and repurposed in response to the virus. Imber-Black (2021, p. 920) concluded that “rituals bent but did not break during COVID-19.” To a large extent, the pandemic has creatively bent our sacred, religious, and spiritual landscape in previously unimaginable ways. How we celebrate a birth, mourn a death, or even eat a family meal have all been disrupted. This calls for reinventing old rituals or inventing new ones. The use of technologically mediated spaces and asynchronous communication to attend and perform rituals and religious ceremonies are key aspects to note (Baker et al. 2020).

We believe that consumers’ revised religious rituals are a potential lens through which we can understand how we respond to such unexpected and threatening situations. We say this because religion and religious rituals can play a pivotal role during such times by providing people with a secure space that helps them cope (Bentzen 2021) and remain positive and healthy (Hart and Koenig 2020). By recreating engagement with and reaffirming the rightness of the sacred, rituals facilitate effective crisis confrontation (Alexander 2012; Shils 1966). In addition, examining religious ritual revision can have significant public policy implications. Heclo (2001, p. 8) appropriately describes the nexus between religion and public policy as “the inescapable coupling” because both are concerned with the observance of values and peoples’ ways of life. Notably, despite secularization theory's claim that religion's role in public life is dwindling (Stark 1999), religion in many cultures continues to play a significant role in the social, cultural, and political realms (Berger 1999).

There are times when it is imperative for traditions and rituals to change because the consequences of failing to do so could be disastrous. The years 2020 and 2021 have provided many examples of how traditional festivals and rituals that did not adapt to the times resulted in superspreader events. Some examples in India include the *Maha Kumbh Mela* festival in Haridwar in 2021 and the Islamic religious congregation *Tablighi Jamaat* at the Nizamuddin Markaz center in New Delhi in 2020. In both cases, the performance of the ritual as normal and the lack of adherence to COVID restrictions resulted in widespread infection. In Israel, the celebration of Purim became a superspreader event in 2020, and crowds panicked at the festival of *Lag B’Omer* in Meron in 2021, resulting in the deaths and injuries of numerous participants. Ironically, in many such cases, rituals of purification became rituals of contamination. Consequently, such risks necessitate alterations to rituals to adapt to public health emergencies.

In this article, we present evidence of changes to some traditional, long-standing Hindu religious rituals during the pandemic. We present four cases: (1) the ritual of worshipping the Mother Goddess *Durga*, (2) the ritual of singing devotional songs or *bhajans*, (3) the ritual of lighting lamps or *diyas*, and (4) the fire rituals or *havans*. Through a systematic discourse analysis of online news articles and YouTube posts, we address the following research questions: How do troubled times like pandemics lead to the revision of rituals? What are the different ways in which these revised rituals are expected to help? How do tradition and invention intertwine in rituals during unsettled times, and what can we learn from such actions to inform future public policy?

To answer these questions, we examine several aspects of rituals: the changes made to ritual elements (ritual artifacts and scripts), who was in control of these alterations (ritual mobilizers and amplifiers), how these modifications were made (morality plays, recontextualization, and the megaphone effect), and what benefits these revisions offered to ritual participants (effectance, expiation of evil and fear alleviation, and community cohesion). Each of these elements provides a distinctive perspective on ritual revisions and on the dynamic and metamorphic nature of rituals meeting cataclysms. We further demonstrate that in times of crisis, new ritual roles, such as ritual mobilizers and ritual amplifiers, may emerge. “Ritual mobilizers” are people who orchestrate the revision. Local artists and sculptors, political figures, religious leaders, singers and musicians, and ordinary consumers emerge as key ritual mobilizers. Further, through their online posts, the press and media as well as ordinary consumers in some circumstances serve as “ritual amplifiers,” or echo chambers who purvey morality plays of good and evil for believers and detractors and help disseminate and diffuse ritual revisions.

The article has six sections. The first offers a brief review of rituals and ritual scholarship in consumer research followed by an overview of Hobsbawm and Ranger's notion of invented tradition. We contextualize the study in the second section with a brief note on the pluralistic nature of Indian society and the Hindu religion. Next, we present the four cases of Hindu religious rituals. This is followed by discussions of our methodology as well as our analysis and interpretations. In the final section, we identify and consider the public policy implications of our study.

## Theoretical Framework

Traditions are the foundation of rituals, and rituals are generally embedded in some sort of belief or tradition. Traditions arise in the stories, legends, and myths that give rise to the ritual. Within religious traditions, these are often laid down in text (e.g., the Bible, the Quran, the *Bhagavad Gita*). Rituals are comprised of the scripts, actors, and symbols that formalize the tradition through certain practices, by appointed leaders. These in turn legitimize and maintain the tradition—the story. As Catherine Bell notes, “Ritual *can* be a strategic way to ‘traditionalize,’ that is, to construct a type of tradition” (2009, p. 124, original emphasis). Therefore, traditions and rituals are intricately linked. In fact, shared rituals are events or practices that become traditions when passed down over multiple generations.

### Rituals

While there are numerous theories and interpretations of both the function and intention of ritual, three notable theorists—Émile Durkheim (1995 [1912]), Arnold van Gennep (1909), and Victor Turner (1969)—have had a marked influence on contemporary ritual theory. There are many types of rituals, including seasonal rituals (e.g., moving from winter to summer pasture), contingent or transformational rituals (e.g., those performed at birth, puberty, and death), rites of affliction (e.g., healing rituals, exorcisms, purification rituals), and divinatory rituals such as prophesy or fortune telling (Turner 1973). Furthermore, rituals may include acts and gestures, such as the greetings and compliments we employ in the micro-social intercourse of daily life (Goffman 1967). As Kertzer (1988, pp. 131–32) notes, “Rituals do have value for many people who otherwise feel impotent before the powers that rule over them. The value of rites is psychological; they reduce people's anxiety level and give them the healthier impression that they do have some control over their lives.” However, rites of passage or transitional rituals, such as birth, initiation, wedding, graduation, and death, which epitomize a person's transition from one position to another, also serve vital social functions, such as the changes caused to the ritual participants’ social status (Rook 1985). Importantly, rituals are symbolic practices that unite people and “create an alliance, a wholeness, a community” (Han 2020, p. 6).

### Rituals and Consumer Research

Colonial-era anthropological and sociological research on rituals regarded them as the practices of primitive savages, viewing such activities purely through the lens of superstitious beliefs and customs (Rook and Levy 1983). This was part of the colonial project in which European colonizers attempted to justify their cooptation and treatment of indigenous others as barely human. However, Rook (1985) and McCracken (1986) have offered a more inclusive view of rituals, as

a type of expressive, symbolic activity constructed of multiple behaviors that occur in a fixed, episodic sequence, and that tend to be repeated over time. Ritual behavior is dramatically scripted and acted out and is performed with formality, seriousness, and inner intensity. (Rook 1985, p. 252)

an opportunity to affirm, evoke, assign, or revise the conventional symbols and meanings of the cultural order. (McCracken 1986, p. 84)

Notably, rituals are often performed without giving much thought to the reasoning that allows access to the sacred (Belk, Wallendorf, and Sherry 1989). Consumer studies of rituals have focused on behaviors linked to sacred experiences and symbolic acts (Holt 1992). This research has been multidisciplinary and multilayered, giving the study of ritual its energy (Stanfield and Kleine 1990).

The term “ritual” has been used in consumer research to encompass a broad spectrum of behavior, from the most intimate and private to the most shared and public (Gainer 1995). Indeed, the power of ritual can turn mundane spaces into lively public spaces of interaction (Bradford and Sherry 2015). Given the potential of ritualization to explain many facets of consumer culture, researchers have extensively investigated ritual and made significant contributions to several topics such as identity, gift giving, and marketplace development. For example, ritual studies have considered the fate of a person's identity project after death (Bonsu and Belk 2003), observed gift giving through the lens of moral and market economy logics (Weinberger and Wallendorf 2012), examined the role of dress in collective identity shifts (Chaney and Goulding 2016), considered Thanksgiving Day as consumer discourse on U.S. consumer culture's categories and principles (Wallendorf and Arnould 1991), analyzed the salience of retailers’ language use to facilitate marketplace formation (Otnes, Ilhan, and Kulkarni 2012), deconstructed the management of symbolic boundaries (Weinberger 2015), and explored the functions of the neotribal and cocreated ritual experiences of clubbing (Goulding and Shankar 2011; Thornton 1996).

Importantly, rituals are also subject to change and revision. In some cases, these changes may be instigated by television, advertisers, and retailers. Television has been found to mediate and change family mealtime rituals, including “eating locations, mealtime conversations, and eating times” (Chitakunye and Maclaran 2014, p. 16). Advertising has also led to shifts in consumption rituals, causing “certain cultural artifacts to become part of the status quo,” such as pumpkin pie, turkey, and cranberry sauce at Thanksgiving (Cross, Harrison, and Gilly 2017, p. 476). While many families feel that Thanksgiving is “universalistic and unchanging,” there are considerable disparities in the ritual elements (such as meal preparation) across ethnicities and classes (Wallendorf and Arnould 1991, p. 23). Advertising also conveys how things designated for specific ceremonial uses in wedding rituals are reconfigured in new constellations (Otnes and Pleck 2003; Otnes and Scott 1996). Gift registry rituals that were initiated in the nineteenth century with jewelers competing with department stores grew into ritualized practices by the mid-twentieth century. Here, retailers acted as ritual “orchestrators” by offering critical resources to ritual participants (Bradford and Sherry 2013). Long-practiced rituals might change depending on ethnicity and culture. For instance, Americans’ holidays and life cycle rituals have evolved from carnivalesque celebrations outside of one's home and with status and wealth displays to both indoor and outdoor celebrations, acknowledging vital aspects such as family, women's roles, and ethnic group consciousness (Pleck 2000). Intercultural weddings also offer a rich context in which to explore ritual modifications. In contrast to mainstream monocultural weddings, intercultural weddings necessitate altering rituals to accommodate two different cultures and religious traditions (Leeds-Hurwitz 2002).

Changes in the meanings and functions of rituals often echo “changes in the national economy, social values, consumer ideology, and gender roles and power relationships,” as shown in Minowa, Khomenko, and Belk’s (2011, p. 51) study of gendered gift-giving rituals in Japanese society. Rituals are “subject to life cycle forces,” and they are sometimes segmented for smaller audiences (Rook 1985, p. 255). Rook (1985) exemplifies the resurgence of ethnic practices within the old model of the American “melting pot” to support this argument of ritual change. While the aforementioned research gives some insight into ritual modifications, there is a dearth of a comprehensive study of changes in religious and public rituals during crisis periods, such as the COVID-19 pandemic, when government policies necessitate ritual adaptations. These ritual modifications, which result in “temporary” invented traditions, may help us understand how consumers react to uncertain times, how rituals are altered, who controls these changes, and who benefits. Our research focuses on these concerns. We next turn to the meaning of traditions and, more importantly, invented traditions.

### The Invention of Traditions

“Tradition,” in simple terms, means any artifact, belief, image, practice, or institution that has been handed down or transmitted from the past to the present (Shils 1981). In anthropological literature, tradition often refers to a time-honored custom. This perspective, however, is not without problems. It suggests that traditions are etched in stone and therefore immutable. Conversely, it has been argued that the structure, details, and interpretations of traditions are subject to change over time and place (Bell 2009). Their alterations can often help us understand transitions and disjunctures (Shoham 2014). Moreover, during times of upheaval and widespread disruption, traditional practices and inventions may coalesce. These convergences may often be transient and short-lived, as with changes surrounding wedding and funeral rituals. Nonetheless, given these changes to the structure and function of the tradition, it is important to investigate these temporary alterations. Through the revision of rituals, as we see in our cases and analyses, traditions are used or bent to suit current purposes.

According to Hobsbawm (1983, p. 1), “invented tradition” refers to “a set of practices, normally governed by overtly or tacitly accepted rules … of a ritual or symbolic nature, which seek to inculcate certain values and norms of behavior by repetition, which … implies continuity with the past.” In “invented traditions,” customary practices are often “modified, ritualized and institutionalized,” and new symbols and devices are created (Hobsbawm 1983, pp. 6–7). Such invented traditions are often reactions to changing circumstances and they may conveniently weld onto old traditions. That is, rituals that invent traditions may include fixed actions and acts that mirror changes in society (Bell 2009).

Sometimes, long-held rituals are *intentionally* reinvented. In some societies, such as the former Soviet Union, rituals functioned as instruments for “cultural management.” Rituals and ceremonies were consciously created and revived to inculcate Soviet socialist values into the daily lives of the population (Lane 1979, 1981). Other examples of the intentional reinvention of traditions include the Christian calendar additions of All Saints’ Day on November 1 (in 731 ce) and All Souls’ Day on November 2 (in 1006 ce). These were attempts to supplant the focus on Halloween (which was full of anti-Christian images of ghosts, witches, hobgoblins, and the devil) and its Celtic progenitor celebration, the Samhain festival (which emphasized the return of the dead and may also have involved human sacrifice) (Belk 1994, pp. 110–11). Accordingly, the meaning of ritual celebrations can be challenged successfully or unsuccessfully and can be temporary or permanent.

In contrast to the deliberate and imposed reinvention of traditions, natural emergent change may also lead to traditions being reinvented. One example of such naturally evolving reinventions is the shifting focus of the American Halloween ritual, from children's “trick-or-treating” activities to include adult costume party events (Belk 1990). Christmas celebrations have also undergone organic changes. Modern Christmas festivities, which symbolize and center on family, gift and card exchange, shopping, Santa Claus, and Christmas trees, are largely Victorian reinventions (see Belk 1989; Moore 2014). Using artifice and creative reformulation, traditions are tweaked, with just enough of the past to lend them authenticity and enough of the present to lend them relevance. Nonetheless, Hobsbawm and Ranger’s (1983) “invented tradition” theory is problematic in that it essentializes tradition as authoritative and invention as unapproved and oversimplifies the complicated interplay between continuity and change (Campbell 1993). Taking these problems into account, Campbell (1993) aptly notes, “A more fruitful approach would analyze the dialectic relationship between tradition and invention and examine how they intersect in cultural life” (p. 83). In our focus on Hindu religious rituals, we observe specific ritual modifications that engender a convergence of tradition and invention in cultural life. Before we turn to these issues, we begin with a discussion of the empirical setting of India and the four Hindu religious rituals examined in this study.

## The Empirical Context of India

Pluralistic Indian society, which is home to many religions, ethnicities, beliefs, rituals, traditions, castes, and festivals, serves as the empirical context for our research. People of many religions such as Hinduism, Islam, Buddhism, Jainism, Sikhism, and Christianity live in India. According to the 2011 census, there are approximately 966.2 million Hindus in India, constituting 79.80% of the country's total population. About 172.2 million (14.23%) are Muslims; Christians (27.8 million) account for 2.30%; Sikhs (20.8 million), 1.72%; Buddhists (8.44 million), .70%; Jains (4.45 million), .37%; and others together account for .66%.

A unique feature of India's overall religious ethos is that religion influences all aspects of society, including the secular sphere (Madan 1991). Though the modern political ideology of *Hindutva* views “India as a Hindu nation and defines Indian culture in terms of Hindu cultural values” (Ramachandran 2020, p. 16), Hinduism, whose rituals are relevant to our study, embodies several systems of faiths and traditions in India. This is in part because, unlike other bounded Semitic religions with a central authority and an accepted holy book (e.g., the Bible, the Quran), Hinduism lacks explicit “definitional contours” that could give it a monolithic position (Smith 1998). As a result, many Hindu religious rituals, as our research demonstrates, are unrestrictedly modified depending on its followers’ changing beliefs and circumstances.

## Four Cases of Hindu Religious Rituals: Pre-COVID-19

We focus on four cases of Hindu religious rituals: the worshipping of Mother Goddess Durga during the festival of *Durga Puja*, devotional songs called *bhajans*, the Hindu ritual of lighting lamps or *diyas*, and Hindu fire rituals or *havans*. As we discuss in the following section, elements of each of these four Hindu religious rituals were significantly altered during the 2020–2021 pandemic in India.

### Case 1: The Ritual of Worshipping Mother Goddess Durga

The Indian subcontinent has a strong heritage of worshipping Mother Goddesses in many different forms. The Durga Puja is one popular example, especially in West Bengal, where this research took place. It is an extensive five-day annual festival within the “nine nights” celebration called *Navaratri*. Its focus is the worship of the many-armed warrior Mother Goddess, Durga (Roy 2005). Our emphasis is on the object of one of the many rituals of the festival—worshiping the Durga *protima* or idol, as part of the greater Durga Puja festival. Traditional artifacts held by the ten-armed idol include a conch shell, a bow and arrow, Shiva's trident, a *vajra* or thunderbolt, a half-bloomed lotus, a sword, a mace, Vishnu's *Sudarshan Chakra* or a discus, flames, and a snake. Durga's weapons are normally associated with male Hindu gods (see Rodrigues 2003). These weapons of strength represent Durga's infinite power against evil. The evil that Durga slays with her trident is represented by the buffalo demon, *Mahisa*. While theme-based Durga Puja *pandals* (fabricated structures where the *puja* takes place) are quite famous in Kolkata and other parts of West Bengal, their themes are recalibrated every year, including changes resulting from COVID-19. In a rite known as *Durga Visarjan* (“Durga immersion”), the Durga idol is immersed in holy waters every year, marking the end of the yearly celebration. Every year, following the festival, the *pandals* are dismantled and rebuilt the following year.

### Case 2: Devotional Songs or *Bhajans*

*Bhajans*, which are quite prevalent in Northern India, refer to “vernacular poetical expressions of devotion set to regional music” (Beck 2012, p. 3). The performance of such a “sonic liturgy” is one of the most widespread Hindu musical traditions (Beck 2012, p. 2). One significant Hindu *bhajan* ritual is singing devotional songs in honor of the Mother Goddess. These devotional songs are called *Mata ki Bhente*. During the Hindu festival of *Navaratri* or “nine nights” that celebrate the Divine Mother, *Mata ki Bhente* songs are highly popular. Surprisingly, this Hindu ritual performance of *bhajans* was also adapted by some Indian singers and musicians during the pandemic. Coronavirus *bhajans* subsequently became very popular on social media across the country. For example, in mid-March 2020, during the festival of colors (*Holi*), when the spread of coronavirus was in its infancy in India, one unique Punjabi corona-*bhajan*, described in the following section, trended on social media. Afterward, several similar *bhajans* became popular in various parts of India.

### Case 3: Ritual of Lighting Lamps or *Diyas*

The ritual of lighting lamps or *diyas* is predominantly associated with the Hindu festival of Diwali, when the deities *Lakshmi* and *Ganesh* are worshipped. However, in the Hindu tradition, many propitious ceremonies start with lighting *diyas*. All *pujas* or worship ends with an *aarti* (i.e., offering to the deities) that also involves lighting a lamp. Indeed, a lamp is lit to start most Indian conferences. *Diyas* are also lit as part of the Saturday rituals in many Hindu religious families. The spiritual meaning of a lit lamp is the elimination of negativity and the attraction of divine vibrations (Singh 2015). This key Hindu ritual took on even greater significance during the pandemic and was repurposed to reflect the situation.

### Case 4: Hindu Fire Rituals or *Havans*

Fire, light, and rituals have long been components of festivals. According to “purificatory theory,” fire is a cleansing agent with destructive power, “which blasts and consumes all the noxious elements, whether spiritual or material, that menace the life of men, of animals, and of plants” (Frazer 1983 [1922], p. 841). Several essential Hindu rites of passage, such as initiation, wedding, and cremation, involve fire sacrifices and rituals called *yajna*, *yaga*, *havan*, or *homa* and often involve Vedic chants and recitations (Beck 2012, p. 42). Oblations or sacrifices in the fire rituals involve many items, including food (e.g., rice, grains, linseeds, butter, ghee), wood, and even animals. It is believed that when burned to ashes, these substances are transformed into a powerful medicine conveyed to astral bodies as fragrance (Singh 2015). Although these *yajnas* are traditionally performed during key rites of passage, *havans* are more generally performed to attain particular desires or achieve a sense of well-being. Foremost, *havans* are considered rituals of purification by which performers are freed of their evil thoughts and vices. As in the other three cases, *havans* were reinvented.

## Methods and Data Collection

This research is concerned with examining public rituals that change due to uncontrollable factors, such as the COVID-19 pandemic. In March 2020, the initial impact of COVID-19 in India was first recognized. This dawning realization also happened to coincide with preparations for the Hindu festival of colors, *Holi*. Our data collection began with observations of the changes in the Hindu religious ritual of singing devotional songs that began to address the virus. In the shadow of the pandemic, another ritual invocation event in April—that of lighting *diyas* by Indian citizens—was called for by the Indian Prime Minister (PM), Narendra Modi, as a show of national unity. Subsequently, we observed and noted changes in the festive season (encompassing Diwali, *Navaratri*, Christmas, and New Year [starting in October in 2020]). During the crisis, four cases of religious ritual modifications examined in our research were the most frequently reported ritual changes in the Indian news. In all likelihood, individual rituals (e.g., grooming) and intrafamily rituals (e.g., eating meals) must have also changed as a result of the pandemic. However, the changes in community-based religious rituals examined in this study garnered greater media attention.

The data for our study consist of online news articles, YouTube videos, and comments on these posts. We examined 51 online news articles across 30 online news sources, 52 YouTube videos, and over 7,000 comments (many of which only had symbols such as folded hands and pictures of flowers). As Table 1 shows, the news of ritual revision was covered five times in *India Today* and *Indian Express*; four times in *News18*; three times in *Hindustan Times* and *Times Now*; twice in *The Hindu*, *Business Standard*, *India.com*, *Economic Times*, *The Print*, *The New India Express*, and *ANI News*; and once in *Swachindia NDTV*, *News Room*, *India TV*, *Money Control*, *Republic World*, *Financial Express*, *DNA India*, *NDTV*, *Mint*, *Tribune India*, *Business Today*, *Hindu Business Line*, *DD News*, *Deccan Herald*, *ABP Live*, *National Herald*, and *Zee5*.

**Table 1.** Data Source: Online News.

**No.**	**News Channel**	**Article Title**	**Date Posted**	**Author/Editor**
**Case 1a: Festival of Durga Puja (Durga as Medical Doctor)**				
1	*India Today*	Durga as doctor, Kartik as sanitation worker: Bengal pandal salutes Covid warriors	Oct. 23, 2020	Shreya Sinha and Indrajit Kundu
2	*India Today*	Artist shows Ma Durga as doctor killing coronavirus. Brilliant, says Shashi Tharoor	Oct. 20, 2020	Nishtha Grover
3	*The Indian Express*	Photo of goddess Durga reimagined as a doctor killing ‘coronasur’ goes viral	Oct. 21, 2020	Trends Desk
4	*India.com*	Durga Puja 2020: Goddess Durga Depicted as Doctor Slaying ‘Coronasur’ with Syringe, Shashi Tharoor Calls It ‘Brilliantly Appropriate’	Oct. 20, 2020	Ritu Singh
5	*News18*	Goddess Durga Reimagined as Doctor Slaying Coronavirus in Kolkata Pandal Goes Viral After Migrant Mother	Oct. 20, 2020	Buzz Staff
6	*Hindustan Times*	Durga idol reimagined as doctor killing ‘coronasur’ goes viral, Shashi Tharoor praises it	Oct. 20, 2020	Srimoyee Chowdury
7	*Times Now*	Photos of doctor killing ‘coronasur’ in Maa Durga's avatar go viral	Oct. 20, 2020	Saumya Agrawal
8	*Times Now*	‘Doctors bhagwaan ka roop hain’: Photo of medic in Durga Maa's avatar goes viral	Oct. 17, 2020	Saumya Agrawal
9	*Financial Express*	Durga Puja 2020: Goddess turns doctor, her children depicted as frontline Covid-warriors in viral image	Oct. 20, 2020	FE Online
10	*Money Control*	Ma Durga idol depicting Goddess as doctor killing coronavirus wins Shashi Tharoor's praise	Oct. 20, 2020	Money Control News
11	*Swachindia NDTV*	Goddess Durga Turns Destroyer of Coronavirus in This COVID-Themed Durga Puja, Shashi Tharoor Praises It As, ‘Brilliantly Appropriate’	Oct. 22, 2020	Anisha Bhatia
12	*Republic World*	Kolkata Durga Puja Pandal Depicts Goddess as Doctor Killing Coronavirus ‘the Demon’	Oct. 19, 2020	Aanchal Nigam
**Case 1b: Festival of Durga Puja (Durga as Migrant Worker)**				
1	*The Indian Express*	A Durga puja pandal showcases women migrant workers in place of the goddess	Oct. 22, 2020	Lifestyle Desk
2	*The Indian Express*	Kolkata pandal replaces Goddess Durga idol with migrant workers as tribute to their struggles amid Covid	Oct. 16, 2020	Shashi Ghosh
3	*The Print*	Stop outraging over ‘migrant’ Durga idol. Hinduism in Bengal doesn't need rescuing	Oct. 18, 2020	Debalina Dey
4	*India TV News*	Kolkata Ma Durga idol as migrant worker impresses B’wood stars, fans	Oct. 19, 2020	IANS
5	*ENewsroom.in*	Durga to be worshipped as a migrant worker in many pujas in Bengal	Oct. 19, 2020	Biswajit Roy and Aritra Singha
6	*National Herald*	Kolkata: A unique Durga Puja Pandal pays tribute to migrant mothers, will worship migrant mother as Goddess	Oct. 16, 2020	NH Web Desk
7	*India Today*	Artist behind Kolkata's viral Durga idol says there's a goddess in every migrant mother	Oct. 18, 2020	Shreya Sinha
8	*India Today*	Kolkata: A Durga puja pandal that salutes migrant workers	Oct. 15, 2020	Indrajit Kundu
9	*Hindustan Times*	Durga Puja pandals in Bengal highlight the plight of migrant workers	Oct. 17, 2020	Joydeep Thakur
10	*Economic Times*	Kolkata's Ma Durga idol depicts hardship faced by migrant labourers during lockdown	Oct. 19, 2020	IANS
**Case 2: Devotional Songs or *Bhajans***				
1	*The Hindu*	‘Kitho aaya corona?’: Coronavirus bhajan by singer Narendra Chanchal goes viral	March 16, 2020	The Hindu Net Desk
2	*Business Standard*	Narendra Chanchal's coronavirus bhajan ‘Kitho aya corona’ goes viral	March 17, 2020	IANS
3	*The Indian Express*	‘Kithon aaya Corona…maiya ji’: Bhajan singer Narendra Chanchal's song on COVID-19 has netizens hooked	March 14, 2020	Trends Desk
4	*Hindustan Times*	‘Kithon aaya Corona’: Bhajan on coronavirus has left people with thoughts.	March 14, 2020	Trisha Sengupta
5	*India Today*	Kitho aaya corona? Viral bhajan on Covid-19 has the internet laughing	March 14, 2020	India Today Web Desk
6	*News18*	‘Kitthon Aaya Corona?’ Video of Coronavirus Bhajan Goes Viral	March 17, 2020	IANS
7	*Times Now*	‘Kitho aaya Corona’: Narendra Chanchal's bhajan on coronavirus has taken Internet by storm [VIRAL VIDEO]	March 14, 2020	Saumya Agrawal
8	*India.com*	Trending News Today, March 15, 2020: ‘Kithon Aaya Corona’: Narendra Chanchal's Viral ‘Bhajan’ on Coronavirus Leaves Internet in Splits	March 15, 2020	Ritu Singh
**Case 3: Ritual of Lighting Lamps or Diyas**				
1	*Economic Times*	PM asks India to light candles, diyas on April 5. And Twitter, as always, is divided	April 3, 2020	ET Online
2	*DNA India*	Prime Minister Narendra Modi lights ‘Diya’, leads India in fight against COVID-19	April 5, 2020	DNA Web Team
3	*NDTV*	Light *Diyas* Sunday At 9 pm For 9 Minutes, Appeals PM Modi	April 3, 2020	Deepshikha Ghosh
4	*The Print*	After bartan bajao, PM Modi asks Indians to light lamps, candles as symbols of hope	April 3, 2020	Neelam Pandey
5	*Mint*	PM Modi calls for 9-min blackout on Sunday, urges citizens to light lamps	April 3, 2020	—
6	*Tribune India*	PM Modi's call for lighting ‘diyas’ and candles draws mixed response	April 3, 2020	Vibha Sharma
7	*The New Indian Express*	Millions of Indians respond to PM Modi's appeal; light candles, diyas, turn on mobile phone torches	April 5, 2020	PTI
8	*Business Today*	Coronavirus outbreak: 9-min Diwali! PM Modi says light candles on April 5	April 3, 2020	BusinessToday.in
9	*The Hindu Business Line*	Modi asks people to light lamps on Sunday; Opposition says, ‘stop the symbolism, act’	April 3, 2020	Our Bureau
10	*DD News*	Nation joins PM Narendra Modi's call to light up diyas, candles at 9 pm today in a collective spirit to defeat Coronavirus	April 5, 2020	—
11	*News 18*	‘Light Diyas of Reason not Superstition’: Opposition Slams PM Modi's Appeal to Indians for April 5	April 3, 2020	NEWS18.COM
**Case 4: Fire Rituals or *Havans***				
1	*ANI News*	Locals perform ‘havan’ in Kolkata to eradicate coronavirus	April 29, 2020	—
2	*The New Indian Express*	Hundreds in Bihar perform herbal havan to ‘ward off’ coronavirus	March 18, 2020	Rajesh Kumar Thakur
3	*ANI News*	‘Havan’ in Prayagraj to combat coronavirus	March 6, 2020	—
4	*The Indian Express*	Haryana: To keep corona at bay, villagers take out ‘havan yatra’ on tractor-trolley, touch every street	April 6, 2020	Express News Service
5	*Business Standard*	‘Havan’ in Prayagraj to combat coronavirus	March 6, 2020	ANI General News
6	*Deccan Herald*	‘Panchagavya havan’ can check coronavirus: BJP MP	March 19, 2020	PTI New Delhi
7	*ABP Live*	Jammu and Kashmir: Havan done to save the country and the world from Corona	May 16, 2020	Ajay Bachlu
8	*Zee5*	Locals perform ‘havan’ in Kolkata to Eradicate Coronavirus	April 29, 2020	—
9	*The Hindu*	BJP MLA leads Agnihotra procession to “fight COVID-19”	May 25, 2021	Rishikesh Bahadur Desai
10	*News18 Buzz*	BJP Leader Performs Mobile ‘Hawan’, Blows ‘Shankh’ in Meerut Neighbourhood to End Coronavirus	May 19, 2021	—

Of the aforementioned news sources, 11 (*India Today*, *The Indian Express*, *India.com*, *News18*, *Hindustan Times*, *Times Now*, *NDTV*, *Economic Times*, *The Hindu*, *Business Standard*, and *DD News*) feature in a Reuters Institute Digital News Report (Krishnan 2021) on the most trusted news sources in India. These news sources were ranked according to their brand trust scores and weekly online reach (for details, see Krishnan [2021]). A random search on Google using appropriate keywords (e.g., “Durga as doctor,” “Durga as a migrant worker,” “Corona bhajan news”) led to further news reports of ritual revision during the pandemic. Saturation was believed to have been reached when new data obtained from other news sources appeared to be redundant (i.e., was duplicating the news in previous data; Saunders et al. 2018).

The first COVID-19 national-level lockdown in India was implemented on March 24, 2020, and lasted until the end of May. Lockdown restrictions began to lift in early June and continued through the end of 2020. Thereafter, statewide lockdowns and lifting of lockdowns proceeded based on the state's individual caseload. The first author kept track of the online news articles from March 2020 to May 2021 (Table 1).

To deal with the problematic issues of self-selection and gain a more diverse perspective, we searched video posts on YouTube for related news (Table 2). This helped us understand our observations better and construct a complete, unedited version of the news footage, such as the devotional song we offer as one of the ritual adaptation cases. YouTube viewers’ comment threads offered us another window into social discourse (Laurier 2016). The numbers of unique mentions on YouTube were 13 for the Durga as medical doctor and migrant worker, 16 for devotional songs or *bhajans*, 10 for lighting lamps or *diyas*, and 13 for fire rituals or *havans*. These video posts garnered millions of views and thousands of comments. Most of the Durga Puja posts were made by news channels and a few by individuals; *bhajans* by musicians, news channels, and individuals; and lighting of *diyas* and *havans* by news channels.

**Table 2.** Data Source: YouTube Posts.

**No.**	**YouTube Link**	**Post Date**	**Posted By**
**Case 1a and 1b: Festival of Durga Puja (Durga as Medical Doctor and Migrant Worker)**			
1	https://www.youtube.com/watch?v=U1pGonou5kI	Oct. 18, 2020	Individual
2	https://www.youtube.com/watch?v=ISCAks361BE	Oct. 21, 2020	Facts Academy
3	https://www.youtube.com/watch?v=0t80aFQxlnE	Oct. 18, 2020	Individual
4	https://www.youtube.com/watch?v=n4woMsCVSfs	Oct. 20, 2020	*Oneindia News*
5	https://www.youtube.com/watch?v=zRs-IzTq5iw	Oct. 19, 2020	Aaj ka Funda News
6	https://www.youtube.com/watch?v=y7Eg_baCWq8	Oct. 19, 2020	Smart India Music
7	https://www.youtube.com/watch?v=Ow2MJtb068Q	Oct. 17, 2020	*Kanak News*
8	https://www.youtube.com/watch?v=ftXgb6f7NFc	Oct. 17, 2020	*Hindustan Times*
9	https://www.youtube.com/watch?v=jslk2aldOFQ	Oct. 18, 2020	*The Times of India*
10	https://www.youtube.com/watch?v=i9KtqayWmeo	Oct. 18, 2020	Indiatimes
11	https://www.youtube.com/watch?v=_bjtd91mTn8	Oct. 22, 2020	Individual
12	https://www.youtube.com/watch?v=OtyqN4Ym8-Q	Oct. 23, 2020	TV9 Bharatvarsh
13	https://www.youtube.com/watch?v=xkkKU6TsJGE	Oct. 16, 2020	*ANI News*
**Case 2: Devotional Songs or *Bhajans***			
1	https://www.youtube.com/watch?v=y4lZw9T3KHY	March 22, 2020	Om Bhakti Mantra
2	https://www.youtube.com/watch?v=MVkm9pJaXE0	March 31, 2020	Jinvani Channel (Jains)
3	https://www.youtube.com/watch?v=oinB4IgLM4M	March 16, 2020	Bhakti Sadhna
4	https://www.youtube.com/watch?v=FCZfzSPhbL4	March 13, 2020	*The Times of India*
5	https://www.youtube.com/watch?v=wR_s5nF6D9U	March 30, 2020	Individual
6	https://www.youtube.com/watch?v=E-BBWU4J0HY	April 17, 2021	Ishwar Bhakti Channel
7	https://www.youtube.com/watch?v=W_nHGJiI9Mk	March 28, 2020	Ankit Telecasting
8	https://www.youtube.com/watch?v=or6jiL2CNA4	March 12, 2020	Guru Music Odia
9	https://www.youtube.com/watch?v=zZUlx-UE_Kc	April 27, 2021	Ishwar Sadhna Channel
10	https://www.youtube.com/watch?v=fTRgFChxFwQ	March 25, 2020	SMS Rajasthani
11	https://www.youtube.com/watch?v=Cu6w3GJaZt4	March 23, 2020	Civil Mantraa
12	https://www.youtube.com/watch?v=XINzw9zyfnY	May 29, 2020	Yuki Music
13	https://www.youtube.com/watch?v=DTGc7ZdU8vY	April 19, 2021	Individual
14	https://www.youtube.com/watch?v=pkY3gXtTt_o	May 13, 2021	Wave Music Bhakti
15	https://www.youtube.com/watch?v=2sxRwVVpxL0	June 10, 2020	Sonotek Bhakti
16	https://www.youtube.com/watch?v=iCG6pErzZeo	March 7, 2020	Individual (Rajasthani)
**Case 3: Lighting Lamps or *Diyas***			
1	https://www.youtube.com/watch?v=nnSaE5qP6Os	April 3, 2020	*Hindustan Times*
2	https://www.youtube.com/watch?v=GOnMVja7yuc	April 6, 2020	India TV
3	https://www.youtube.com/watch?v=nbLQcF2hObA	April 5, 2020	*India Today*
4	https://www.youtube.com/watch?v=YezYbXc4F6A	April 3, 2020	*India Today*
5	https://www.youtube.com/watch?v=M6lsBnP24C0	April 5, 2020	*The Economic Times*
6	https://www.youtube.com/watch?v=7BR9-eWxfB8	April 5, 2020	*The Times of India*
7	https://www.youtube.com/watch?v=WglQUfzYvVI	April 5, 2020	Individual
8	https://www.youtube.com/watch?v=k_9eyoWqOh0	April 5, 2020	*The Tribune*
9	https://www.youtube.com/watch?v=mdPnB6hGu-8	March 22, 2020	*The Times of India*
10	https://www.youtube.com/watch?v=KNzIEoX690g	March 23, 2020	*Hindustan Times*
**Case 4: Fire Rituals or *Havans***			
1	https://www.youtube.com/watch?v=o3IwJY7_eNs&t=22s	March 6, 2020	India TV
2	https://www.youtube.com/watch?v=9YctUKSg80Y	March 15, 2020	First Bihar Jharkhand
3	https://www.youtube.com/watch?v=YQQKXAmdCLI	March 17, 2020	Punjab Kesari Haryana
4	https://www.youtube.com/watch?v=wDpV_Mikui4	April 22, 2021	ETV Bharat Bihar
5	https://www.youtube.com/watch?v=79NGWdM_zL4	March 5, 2020	NewsX
6	https://www.youtube.com/watch?v=8y4Mh6iwvT8	March 19, 2020	IBC24
7	https://www.youtube.com/watch?v=4BgmZopNavk	March 18, 2020	Live Hindustan
8	https://www.youtube.com/watch?v=ReUa0uk8zdo	March 16, 2020	Punjab Tak
9	https://www.youtube.com/watch?v=yXV3xGElVRY	May 31, 2020	TV9 Bharatvarsh
10	https://www.youtube.com/watch?v=O3_KPjKR_5Q	March 23, 2020	ABP Ganga
11	https://www.youtube.com/watch?v=0_9×15zIkRM	April 29, 2020	India TV
12	https://www.youtube.com/watch?v=yfgEJTIoiUs18th	May 18, 2021	Oneindia News
13	https://www.youtube.com/watch?v=zHHJTTTwn7k	May 10, 2021	Amar Ujala Punjab-Haryana

The objective of our analysis was to examine cultural representations in the media and how “they make available and thus perpetuate shared meanings” (Willig 2014, p. 342). Therefore, we conducted a discourse analysis to interrogate our data. After conducting an initial open reading of the texts, we applied the approach to discourse analysis proposed by Carvalho (2000). All three authors were involved in the analysis. We began by identifying the main themes of the article, the actors (doing things or being talked about), and the language and rhetoric used. Then, we used a discursive framing technique to figure out the underlying ideas that seemed to drive the construction of the text. Identifying framing devices such as metaphors, exemplars (e.g., historical examples of morality), catchphrases, depictions, visual images, and photographs was key to our analysis at this stage.

Discourse analysis also helped us identify ideological viewpoints expressed in the narrative construction. Our analysis ended with contextual analysis. During this stage, we considered both the texts’ comparative-synchronic (i.e., simultaneous representations of the online news in the same or other news channels) and historical-diachronic (i.e., the temporal unfolding of discourse) aspects. For example, in our first case of Durga idol alterations, we discovered seven online articles in seven distinct news outlets published on the same day (October 20, 2020). All of these stories had headings that said or implied “Durga as doctor killing the coronavirus” (with minor variations). This recurring theme and the story's wide daily coverage on various news channels, are crucial indicators of the events’ prominence. A comparative-synchronic analysis for the other three cases of Hindu ritual revisions discussed in our study also revealed the events’ conspicuousness, evidenced by simultaneous coverage in several outlets. We undertook a historical-diachronic analysis of these instances by looking backward in time as well as following the events as they unfolded over time, as recently as May 2021. When looking for examples of modifications in religious rituals in the past, we discovered several lines of evidence of such ritual changes in the Hindu traditions. In addition, we noticed the temporal evolution of discourses. For example, in the case of fire rituals or *havans*, what began as modest acts to fend off the coronavirus in early 2020, became highly politicized events fueled by Hindu nationalist feelings by mid-2021. We discuss these in more detail subsequently.

## Analysis and Findings

**Figure 1.**
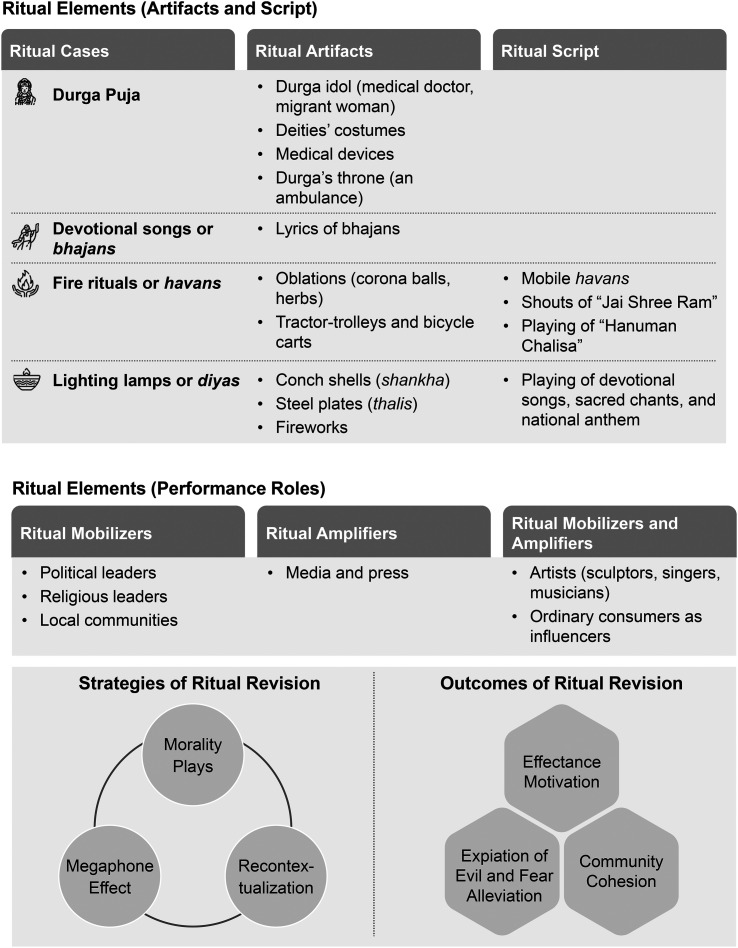
Ritual revision.

In all the four Hindu ritual examples discussed, the two cardinal concepts of “invention of tradition”—continuity and change—are present. During Durga Puja, the long-practiced religious rituals retained continuity with the custom of idolizing and worshipping the Mother Goddess, lighting *diyas* during auspicious days, performing *bhajans* during *pujas*, and enacting *havans* for purification. Even though many religious festivals were curtailed in India due to social distancing requirements, the importance of sociality as a part of the festival/ritual experience encouraged people to continue certain rituals and festivals while adhering to social distance rules. Yet the changes brought on by the virus also led to adaptations and revisions in the rituals themselves. For instance, in some Puja *pandals*, the idol of the Mother Goddess was built around the coronavirus theme, with the Divine Mother depicted killing the *Mahisa* or demon, which in this case was represented as the coronavirus molecule. *Bhajans* were also themed around the virus, while *havans* or fire rituals that mark essential life events were performed to eliminate the pandemic. Likewise, to demonstrate unity against the outbreak, *diyas* were lit. Traditionally, *diyas* are lit during important Hindu festivals or religious occasions. Therefore, the “timeless order” and “established structure” of the Hindu traditions remained part of these revised Hindu religious rituals (see Post 1996, p. 91). However, in each case, either the rituals were repurposed or some parts of the rituals were modified and adapted to meet the changing needs of the time. Next, we discuss the alterations made to the ritual elements, the parties who led these changes, the strategies used to revise the rituals, and the outcomes of these modifications. Figure 1 depicts these aspects.

### Changes in Ritual Elements: Artifacts, Script, and Performance Roles

Rook (1985) identified four broad ritual elements: artifacts, script, performance roles, and audience. Ritual artifacts are goods used in the ritual setting to express particular symbolic messages, and ritual scripts are a sequence of behaviors performed by people who hold significant ritual roles (Rook 1985). A ritual participant's role can be clear or ambiguous; “extensive, limited, or non-existent”; and active or passive (Rook 1985, p. 253). A ritual audience is the group of people who are targeted by a ritual. They can be as specific as an immediate family in mealtime rituals or as broad as the entire COVID-affected humankind, as with the four rituals our study examines. We noticed considerable changes in ritual artifacts in our first two cases and both ritual artifacts and scripts in the latter two. In addition, new ritual performance roles—ritual mobilizers and amplifiers—emerged in our analysis.

#### Changes in the ritual artifacts

##### Ritual of worshipping Durga

**Figure 2.**
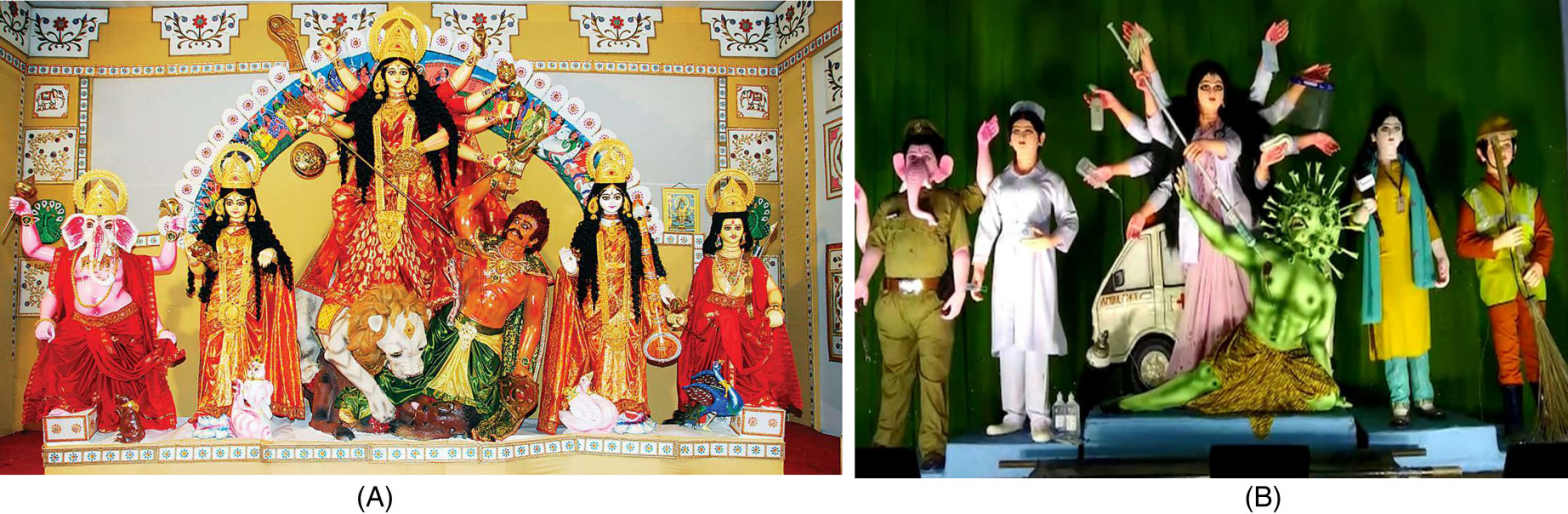
Comparison between Durga idols. (A) Traditional Durga idol and (B) Durga idol during COVID-19.

*Notes*: Source for Panel A image: https://www.dnaindia.com/india/report-durga-puja-2020-petition-filed-in-calcutta-hc-to-stop-celebrations-in-bengal-due-to-COVID-19-2849849. Source for Panel B image: https://www.indiatoday.in/india/story/durga-puja-pandal-bengal-north-dinajpur-tribute-corona-warriors-1734284-2020-10-23.

**Figure 3.**
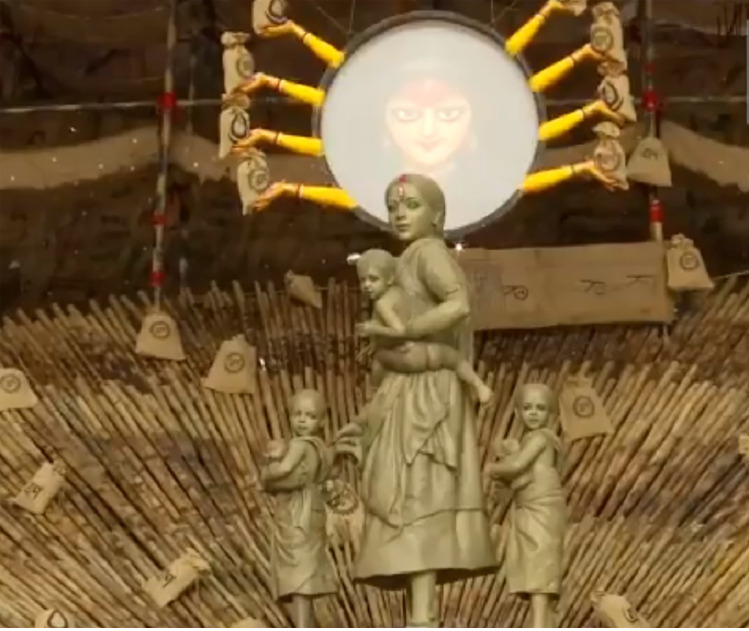
Durga as a migrant worker.

*Notes*: Image source: https://english.jagran.com/trending/puja-pandal-in-kolkata-replaces-durga-idols-with-statue-of-migrant-woman-to-tribute-mothers-workers-amid-covid19-crisis-10018351.

Two themes were prominent in the 2020 Durga Puja *pandals* (see Figure 2, Panels A and B, and Figure 3). The *pandal* shown in Figure 2, Panel B, paid tribute to coronavirus warriors: doctors, nurses, police officers, and sanitation workers (Sinha and Kundu 2020). The theme of a second Puja *pandal* was the suffering of India's migrant workers who lost their livelihood and had to walk, sometimes hundreds of kilometers, to their home villages during the first wave in India (Sinha 2020a). This also became a very popular *pandal* theme, as depicted in Figure 3.

The variations in ritual artifacts observed were in the Durga idol, transformed into either a medical doctor or a migrant worker. They also include the addition of medical equipment in Durga's ten hands and the design of her throne. In Figure 2, Panel B, the goddess is depicted as a doctor killing the coronavirus. In this case, the coronavirus replaced the head of the anthropomorphized buffalo-demon *Mahisa* in a striking contrast to the traditional Durga and *Mahisa* in Figure 2, Panel A.

We also see that Durga is slaying *Mahisa* not with her traditional trident but a giant syringe of coronavirus vaccine. Furthermore, in place of the lion that usually acts as her vehicle, Durga is seen riding an ambulance; instead of her traditional weapons, Durga is depicted carrying medical instruments such as a saline drip and a pulse oximeter, which are used in treating COVID patients. The other deities worshipped as a part of the 2020 Durga Puja festival are also portrayed in roles befitting the COVID-19 heroes theme. These deities wear the uniforms of a police officer and a nurse, whereas the two others are dressed as a journalist and a sanitation worker.

The Durga Puja shown in Figure 3 pays homage to the millions of Indian migrant workers who faced multiple hardships in returning home during the pandemic. As one of the most vulnerable groups in the economy, millions of Indian migrants engaged in “casual labor contracts” were suddenly displaced in the initial lockdown (Sengupta and Jha 2020). The sufferings of these migrant workers were captured in one of the Durga portrayals as a migrant mother (see Figure 3).

Without any jewels and adornments, the plain-looking Durga *protima* idol symbolizes the misery shared by these millions of Indian migrant workers. Both of the Durga Puja *pandals* discussed symbolize a world affected by COVID.

##### Devotional songs or *bhajans*

As noted previously, *bhajans* are devotional songs sung in praise of deities. However, the corona-*bhajan* we cite as an example bears well-crafted messages for the audience and differs from the traditional *bhajans* (*The Hindu* 2020). The ritual artifact (lyrics) of the *bhajan* in this case were coronavirus-themed (https://www.youtube.com/watch?v=ml9XWt0PIAk) and mainly offered advice on safety measures to avoid infection. The *bhajan* also spreads social awareness by mentioning covering the face with masks and using hand sanitizers. By incorporating some element of humor in the lyrics, the *bhajan* restores the audience’s faith that the Mother Goddess will relieve everyone's sufferings. *Bhajans* often have repetitive lyrics that reinforce the devotion. The words “*Kitthon Aaya Corono*” (“Where Did Corona Come From”) and “*Nassega Corona*” (“Corona Is Going to Run Away”) were repeatedly used in this *bhajan*. Collectively singing such devotional songs, which generally have a call-and-response musical structure, also dilutes the dominant dichotomy of performers and audience (Kannan 2016), thereby further reinforcing the ritual actors’ collective strength in the fight against COVID-19. A slew of additional corona-themed *bhajans* gained traction in different regions of India.

#### Changes in the ritual artifacts and script

##### Ritual of lighting *diyas*

The Indian PM, Narendra Modi, urged Indians to light *diyas* for nine minutes on April 5, 2020, to show solidarity in the battle against coronavirus. Heeding the PM's request, Indians turned off all the lights in their house and lit *diyas* for nine minutes. This act, however, was not restricted to lighting *diyas* and included much more than the PM had requested. The performance of the ritual of lighting lamps involved new artifacts and scripts. For example, in some places, lighting *diyas* was paired with the blowing of conch shells called *shankha*; the beating of *thalis* or steel plates; lighting fireworks; blowing whistles; and playing the national anthem, sacred chants, and devotional songs (*The New Indian Express* 2020). Notably, both blowing of *shankha* and beating of *thalis* are considered sacred Hindu practices.

The lighting of *diyas* and candles symbolized the dismantling of the darkness produced by the outbreak (BusinessToday.in 2020). In addition, some speculated that the PM's choice of timing had some “astrological” reasoning (Sharma 2020). The number nine has broad significance in Hinduism. For example, *Navaratri* is the nine-day Hindu festival. *Navras* or the nine *Rasas* (emotions) are a significant aspect of the Hindu *Rasa* theory. *Navgraha*, or the nine planets, find a vital discussion in Hinduism. And *Navratna*, or the nine-stones, define Vedic astrology.

##### Fire rituals or *havans*

Since the global COVID outbreak, many Indians have been seen performing *havans* to eradicate the pandemic. In one video, people in West Bengal perform *havans* and sacrifice red symbolic coronavirus balls to the fire (*ANI News* 2020). During the *havan*, one of the performers is shown holding a placard with “*Corona-mukti yagya*” (where “*mukti*” means free and “*yagya*” means sacrifice; thus a freely given sacrifice) written on it. Such fire rituals have been performed in numerous Indian states and villages. Another innovative “herbal *havan*” performed in Bihar involved oblations of 60 different types of herbs to purify the environment in the hope of eradicating the virus (Thakur 2020). In other locations, a proposal was made to perform a unique *panchagavya havan* (“*pancha*” means five and “*gavya*” means a cow) involving the five substances obtained from the cow (urine, milk, ghee, curd, and dung) to eradicate COVID (*Deccan Herald* 2020).

While these examples show changes in ritual artifacts in the form of oblations, there are many more examples of changes in ritual scripts with simultaneous changes in artifacts. “Mobile” *havans* taking place on tractor trolleys and carts have become popular. A *havan yatra* (where *yatra* means trip) was performed in a northern Indian Haryana village on a tractor-trolley that traveled through all the village streets intended to purify the atmosphere (*The Indian Express* 2020b). Such instances of mobile *havans* were not restricted to villages, however. Some city-based mobile *havans* were influenced by nationalist Hindutva [Hindu nationalist] sentiment and led by Bharatiya Janata Party (BJP, the ruling party) leaders. For example, in May 2021, a BJP Member of the Legislative Assembly performed an Ayurveda-based “Agnihotra havan” (“healing fire *havan*”) in Belagavi in the state of Karnataka. This mobile *havan* involved burning herbs to purify the air of the coronavirus droplets (Desai 2021). Another BJP leader led a mobile *havan* on a bicycle cart in Uttar Pradesh's Meerut district. The ritual script underwent two significant alterations. One was the shouts of “*Jai Shree Ram*” (“Hail Lord Ram”) from the ritual participants, and the other was the playing of *Hanuman Chalisa* on speakers in the background (News18 Buzz 2021). Hanuman Chalisa is a devotional song dedicated to the monkey-god Hanuman, a devotee of Lord Ram (Rama) himself.

#### New ritual performance roles: Ritual mobilizers and amplifiers

##### Ritual mobilizers

In the examples discussed, various parties—whom we call “ritual mobilizers”—catalyzed the shifts in religious rituals leading to their revision. These include political leaders, local committees, artists and sculptors, religious leaders, singers, and musicians, as well as ordinary consumers. During the ongoing pandemic, these ritual mobilizers have attempted to operationalize the ritual revision in distinct ways.

The local *puja* organizing committees play a significant role in determining the *pandal* theme and the overall execution of the Durga Puja. They are also often responsible for choosing the makers of the Durga idol and the *pandal*. Other stakeholders, such as the *pandal* and idol designers, also contribute their creative ideas. Amid the pandemic and the increase in the nation's infections, the government's objective was to ensure that citizens obey social distancing norms and preserve the peace, as well as remain united in the common fight against the outbreak. To this end, in this time of crisis, PM Modi invoked the Hindu religious ritual of lighting lamps or *diyas*, ostensibly to create positivity and solidarity. By producing devotional songs or *bhajans* based on the coronavirus theme, musicians have also played an essential role in ritual revision. Finally, to combat the virus's spread, *pandits* (seers) who perform *havans* and local community members participating in these rituals helped adapt, accept, and accelerate the modified rituals.

Regarding ritual revision, even micro-mobilization efforts on the part of individuals and small groups elicited intense emotional reactions. Ordinary women, affiliated with a middle-income welfare association in Bihar, were spotted performing group herbal *havans* that subsequently made the headlines (Thakur 2020). Similarly, locals in West Bengal performing coronavirus eradication fire rituals became popular online (*ANI News* 2020). “The megaphone effect” (McQuarrie, Miller, and Phillips 2013), which we discuss in the subsection on strategies of ritual revision, explains some of these instances. As we have discussed, ritual mobilizers are those who created, orchestrated, or led the ritual revisions. As such, they are different from ritual amplifiers, who help diffuse the ritual revision.

##### Ritual amplifiers

Much work regarding the intersection of religion, culture, and the media has been triggered by digital technology's explosive growth over the last few years. Prohibition of large gatherings and the imposition of social distancing rules have further enhanced the significance of the media in our lives. The press and the internet have played a pivotal role in circulating news about religious rituals, producing emotional responses. They have played an important role via various cultural agents, enabling widespread mobilization (Downing 2008). News channels frequently reported the endorsement of ritual changes by Bollywood celebrities and cricketers (*Economic Times* 2020). The media functioned as ritual amplifiers rather than passive message conduits. The media gather, select, reject, and recount narratives of ritual modification, frequently generating “the myth of a common focus, [as] a center of concern” (Hurd 2014, p. 298). In this way, the media also legitimize ritual revision. The role of the media in defining control and encouraging involvement is blatantly overt in some cases, such as the BJP leaders’ mobile *havan* rituals suffused with nationalist Hindu sentiments.

In addition to the press, individual consumers have also acted as amplifiers in ritual revision by capturing events, posting them online, and commenting on them. Reportedly, the photo representing the Durga idol as a warring medical doctor was initially published on Facebook (Chowdhury 2020). It was reposted numerous times on platforms such as Reddit and WhatsApp (*The Indian Express* 2020a). Furthermore, the coronavirus *bhajan* that we described gained popularity after being shared on Instagram by an Indian comedian (*Business Standard* 2020). In other cases, a group of Rajasthani women's online posts of corona-themed *bhajans* made headlines (Sengupta 2020). In the *bhajan*, the women are shown singing a festive song with the words “*Corona bhaag jaa, Bharat me tharo kain kaam re*,” which translates to “Corona run away, there is no work for you in India” (Agrawal 2020). It follows that some ordinary consumers served as mobilizers (e.g., seers performing *havans*), others as amplifiers (e.g., the Indian comedian disseminating news of the revised *bhajan*), and still others as both mobilizers and amplifiers (e.g., the individual singers revising the *bhajans* and posting them on YouTube).

#### Strategies of ritual revision

##### Morality play: Good versus evil

The recurring theme of good versus evil has a strong foundation in many religious belief systems (Buchholz and Mandel 2000). “The whole of religious life gravitates around two opposite poles” of the good/benevolent and the evil/impure (Durkheim 1995 [1912], p. 413). The idea of “evil” exemplified by the intricate divine–demonic (*daivi*–*asuri*) dialectic is one of the foundational aspects around which Hinduism as a religion is structured. Concepts of *dharma* (morality, justice, and order) and *adharma* (immorality, injustice, and chaos) are frequently mentioned in the Hindu sacred text the *Bhagavad Gita*, which includes several instances where an avatar or the incarnation of the divine emerges when *dharma* collapses. For example, during *pralaya*, catastrophic geological phenomena in Hindu theology similar to the chaos created by the coronavirus pandemic, it is believed that “Lord Vishnu, the saviour, emerges into this world in the form of ‘*avatar*’ (incarnation)” to save the world from annihilation (Chandrashekharam 2007, p. 29).

We find that a new narrative was developed around coronavirus as the evil enemy of humankind in the four examples discussed previously. In the cases of the Durga Puja and the coronavirus *bhajan*, the coronavirus was given human-like qualities to further this good-versus-evil narrative. The significance of coronavirus anthropomorphism resonates with a recent study by Wan, Kulow, and Cowan (2022), which shows that people are more likely to use protective gear such as masks and gloves when diseases are anthropomorphized. People bestow viruses with human-like qualities and as something tangible rather than invisible, an entity that can be fought with the help of modern medicine. This was explicit in the Durga Puja *pandal*. Evidence of a battle is also present in the *bhajans* songs where the virus is anthropomorphized as something in retreat. Such adversarial warfare motifs are common in the Indian classics the *Bhagavad Gita* and the *Ramayana* as well as in ancient Vedas. As the Durga Puja in Figure 2, Panel B, aptly enacts, the coronavirus takes the place of the devil figure *Mahisa*. The battle that is pitched is between all of humanity and an evil disease.

Overall, the good-versus-evil theme implicit in ritual revisions and manifested through anthropomorphizing the virus represents the battle for a coronavirus-free world. This theme of good versus evil also appeared in the lighting of *diyas* and lamps by the Indian people. Lit *diyas* marked the destruction of darkness cast by the evil virus. In some Indian states, the purpose behind performing the Hindu fire rituals or *havans* was also the removal of coronavirus, which was considered an evil force bringing harm to humankind. Here, the theme of morality plays that pits good against evil predominates. The evil in one instance was transfigured as the red corona balls consumed by the fire in a triumph of good (health).

##### Recontextualization

By “recontextualization,” we mean the process by which religious rituals performed in one traditional context are revised and repurposed for another context. As mentioned previously, the Indian PM urged citizens to light *diyas* for nine minutes on April 5, 2020. Significantly, this ritual of lighting *diyas* is a cardinal element of the most important Hindu festival, Diwali, which is variably in October or November. We interpret the PM's invocation of the *diya* or lamp-lighting ritual and the mobilization of the population to participate in the solidarity performance as “recontextualization.” This entailed borrowing the religious ritual of lighting *diyas* and its associated symbolism and using them in a new context. The outbreak created a situation in which the opposition party and the public began challenging the ruling BJP government's management in India. The BJP government recontextualized the religious ritual to control the unprecedented situation posed by the pandemic, exacerbated by the opposition's attacks and the general loss of public faith in the administration. A small fraction of people expressed their resentment toward the ritual recontextualization in the comments to the YouTube posts^1^:

Do things that make sense, help the poor, provide them food and SHELTER!

Here [are] poor migrants on road without food and transport.

The topics where “Light” has to be shed upon are…How will daily wagers survive?…How is PMCare fund being used?…When will all Doctors get PPE?…Why is India testing so slowly?

However, millions of Indians heeded Modi's appeal to turn off lights and light *diyas* for nine minutes, visibly demonstrating the success of this recontextualization strategy (*The New Indian Express* 2020).

Notably, the strategy of recontextualization was not limited to religious rituals. It also included Hindu myths. For instance, while making the request, the PM also invoked tales from the Hindu epic *Ramayana* to warn the population against violating social distancing rules. For instance, he urged the people not to cross the “*Lakshman Rekha*” (Ghosh 2020). *Lakshman Rekha* was the line drawn by *Lakshman* in his house to safeguard his sister-in-law *Sita* while he went in search of his brother *Rama*. *Sita* was eager to help *Ravana*, the demon king who disguised himself as a mendicant. She crossed the line and was kidnapped by him. In another instance, Modi also referred to societal distancing as the “*Ram Baan*” (“the Arrow of Lord *Rama*”) (Pandey 2020). With this Supreme Arrow, *Ram* later killed the demon king *Ravana*. PM Modi orchestrated the new ritual of lighting lamps in solidarity during times of crisis with great success, appealing to the Indian people's culturally ingrained religious beliefs.

Another example of recontextualization was the performance of fire rituals or *havans*. As previously indicated, fire rituals are employed in various Hindu rites of passage, and they are also used to produce a general sense of well-being. In India, fire rituals were performed in numerous towns and villages in the hopes of eradicating the pandemic (Thakur 2020). While the Indian PM's invocation of the lamp-lighting ritual was the only top-down call for ritual revision, the recontextualization of *havans* seemed to be more organic, with the exception of a few instances in which local political figures began these rituals (Desai 2021; *News18* 2021).

##### The megaphone effect

The “megaphone effect” occurs when ordinary people with no professional expertise or institutional or familial position post content online and gain “a mass audience of strangers” (McQuarrie, Miller, and Phillips 2013, p. 137). This effect may be more visible during times of crisis when consumer-to-consumer engagement on social media increases dramatically (Azer, Blasco-Arcas, and Harrigan 2021). In the discussion on ritual mobilizers, we noted multiple instances of ordinary consumers, with no institutional position or professional competence, posting videos of performing *bhajans* or *havans*. One of the instances was singing of a *Holi* song by a group of Rajasthani women when they were heard urging corona to leave the country (Agrawal 2020; see also https://www.youtube.com/watch?v=iCG6pErzZeo). Another example is the article about reimagining Durga as a medical doctor, supposedly shared over 69,000 times after being posted by a Facebook user, with photographs shared on platforms such as Reddit and WhatsApp and generating headlines in the news (*The Indian Express* 2020a).

With no “prior institutional mediation,” these consumers were able to “grab the megaphone” for themselves (McQuarrie, Miller, and Phillips 2013, pp. 137, 140). After these consumers had garnered a following, the mainstream media accessed and leveraged these posts, playing, in the process, the role of ritual amplifiers.

Furthermore, referring to Nguyen and Dolbec’s (2020) work, we made two distinct observations. These posts appeared to be less strategic and intentional in some cases, such as fire rituals or *havans*. This means that the people who were performing these rituals were not necessarily attempting to develop a brand or position for themselves. Conversely, many of the *bhajan* singers and music channels positioned themselves as “digital entrepreneurs,” thus capitalizing on the crisis. This became evident from some of their YouTube posts (see, e.g., https://www.youtube.com/watch?v=or6jiL2CNA4, https://www.youtube.com/watch?v=2sxRwVVpxL0).

### Outcomes of Ritual Revision

These revised religious rituals seem primarily designed to provide Indians with hope of transformation during the pandemic. The fantasy aspects created by lighting lamps or *diyas* were a symbolic dismantling of the darkness—of afflictions induced by the pandemic. The following are two comments on the YouTube posts:

The universal luminisence [sic] will create a massive energy of agni the fire which will destroy the effects of the virus.

The diya is for positive energy and bringing everyone together. The universe gets this energy and people get together with their families for a single cause.

The first quote here alludes to the spiritual powers of the Hindu fire god (*Agni*) while also demonstrating people's effectance motivation to modify certain aspects of the environment—for example, producing the fire that would destroy the virus. We discuss the concept of effectance motivation subsequently. The second quote suggests community cohesion and a sense of togetherness that the ritual changes in lighting diyas or lamps generated. This feeling of solidarity was also evident in the comments present in news articles. For example, in the context of lamp lighting, an Indian citizen commented, “Crores [tens of millions] of people are doing it at the same time. It has connected all of us in one thread. We stand shoulder-to-shoulder in these times of crisis. We are one. India is one” (*The New Indian Express* 2020). Similarly, the oblations of corona balls were symbolic of the destruction of evil and liberation from current woes (*ANI News* 2020). Consequently, these revised ritual practices represented rituals of transformation (see Belk and Costa 1998). They also provided an opportunity for self-renewal and temporary community cohesion. As such, participation in these rituals functioned as rites of intensification of community bonding (Belk and Costa 1998).

The purpose of many of the revised rituals (e.g., lighting lamps, performing *havans*) was not only to fight the virus or to cause good to triumph over evil. They were also a means of providing people with a sense of control and efficacy, a way to foster a belief that something was indeed being done: “It's not about politics and religion but about the collective intention to move from fear to being hopeful” (comment to a YouTube post). Our data demonstrate the performance of *havans* using herbs and disinfectants to purify the environment. As mentioned in one of the news articles, “The main objective behind organizing the havan was to eliminate viruses through environmental impacts of herbal Havan” (Thakur 2020). Reportedly, *havans* were expected to generate “a medicinal atmosphere” and lower “the bacterial count” (*The Indian Express* 2020b). Performances of herbal *havans*, along with the quote about producing fire (or Agni) to eliminate the virus's effects, suggest a sense of effectance motivation. Effectance motivation, or “the desire for effective interaction with the environment,” involves focusing attention on a specific aspect of the environment and directing actions that may have an impact on it (White 1959, pp. 317, 322). It is the sense that “we’re doing something about it; not just sitting around.” White's (1959) concept of effectance motivation taps into several aspects of motivation, including a desire to impact the environment, to deal effectively with the environment, and the feelings of efficacy that arise from these actions (Harter 1978, p. 35). The analysis of our cases reveals ritual participants’ displays of effectance motivation in their ability to bring changes to the environment.

Studies have also demonstrated that anthropomorphizing satisfies our effectance motivation (Waytz et al. 2010). Consumer research on anthropomorphism affirms that consumers can build ties with nonhuman objects (e.g., Hart, Jones, and Royne 2013), including anthropomorphized smart devices (Schweitzer et al. 2019). Anthropomorphic ideas are also prevalent in several world religions. Durkheim’s (1995 [1912], p. 62) observation that “we tend instinctively to conceive all things in our own image, that is, as living and thinking beings” is of great relevance in two of the cases we offer. People perceive viruses with human-like qualities and as something tangible rather than invisible. Although, in the cases of the Durga Puja and *havan*, the anthropomorphic image of the virus was visual, anthropomorphism is also evident orally in the *bhajan*. The lyrics with the words “Nassega Corona” (i.e., “the corona will run away”) suggest that the virus has form, will be defeated, and will flee.

Instead of being merely “expressive,” the symbolic acts of lighting *diyas*, singing COVID *bhajans*, and creating coronavirus-themed Durga Puja *pandals* were used purposefully to “transform, address, or otherwise influence society” (Santino 2009, p. 11). Other rituals have other purposes, but these were designed to create a sense of calm and reduce fear. Such ritual revisions are not solely confined to the current pandemic. They also derive from the need to assuage the fears of people in times of crisis. They are also acts of unity and moments of light-hearted escape. For instance, the experience of audiences’ times together in the *bhajan* rituals and viewing Durga Puja *pandals* help them socially “‘tune-in’ to one another, to share an inner state of consciousness” (Spickard 1991, p. 197).

Broadly speaking, the production and presentation of these public symbolic acts serve to reform society and its members’ attitudes toward the pandemic and render its features as “ritualesque.” This involves “the performative use of symbols to effect social change” (Santino 2009, p. 25). As we have discussed, singers performing *bhajan* rituals and those undertaking mobile *havans* have consciously revised the traditional rituals to alter public attitude during the crisis. The revised Hindu religious rituals that we discuss, therefore, are “ritualesque” in nature. The migrant Durga idol (Figure 3) also symbolizes the suffering shared by millions of Indian migrant workers. This version of the revised Durga idol also supports Kelly and Kaplan’s (1990) view based on the theory of alterity (self/other) that rituals can become one of the many ways to build and communicate self/other relationships. In this case, the expression is one of empathizing and heroizing rather than distancing.

## Discussion

It has been recommended that given the multidimensional nature of uncertainty created by pandemics such as COVID-19, it is essential to ensure coordination across various systems such as public health, government, the economy, and environment at local, state, and national levels when creating public policies (Mende and Misra 2020). Wiener, Ellen, and Burton (2020, p. 372) warn that “COVID-19 will likely have further multidimensional ramifications for marketing and public policy for many years to come.” Heeding such words of caution and focusing on Hindu rituals in India, we highlight the link between religious rituals and public policy observed during a time of national and global crisis.

Drawing on online news content and YouTube posts, our observations focused on the Durga Puja festival, Hindu devotional songs or *bhajans*, lighting lamps or *diyas*, and fire ritual or *havan*. In the wake of the current outbreak, these rituals have undergone profound revisions to address the coronavirus theme. Moreover, they have been revised in such a way as to leverage the power and familiarity of past traditions, primarily to avoid panic, lessen fears, and create feelings of efficacy and unity. An understanding of these revised rituals informs our understanding of how ritual revision can be one of the many ways to navigate the challenges created and spread awareness during turbulent times. We provide some recommendations based on our study of Hindu rituals, but these findings may be adapted to fit the rituals of other religions, so long as this core message is retained in a form that the audience can identify with.

### Involve Diverse Social Actors

Our consideration of the four cases of ritual revision suggests that they not only offer a compelling case, showing how traditions are modified, but also provide a vivid opportunity to examine how these transformations have taken place by involving several social actors. While faith leaders are pivotal to the acceptance of the message, seemingly other trusted members of the faith community (artists, poets, performers, singers, storytellers, etc.) may also play a part in strengthening the appeal and acceptance of the message. The stakeholders involved in ritual revision we discuss include ritual mobilizers and ritual amplifiers. It appears that in some cases, local artists, such as singers, musicians, and sculptors, may have a stronger regional presence and credibility than national governments. Our findings suggest that prominent community members may be more influential in disseminating information and mobilizing people during times of crisis. Bearing this in mind, community-based associations made up of the various social players, some of whom we have listed, can be formed whenever practicable. Film actors, sportspeople, and regional and national gurus might also be employed in the future. It may be beneficial to provide artists with government funds, support from health agencies, and company sponsorship for disseminating health-related information.

### Create Faith in Science Through Engaging Stories

While there is this growing convergence of faith and science around the globe to combat the pandemic, we suggest that communication can be more effective if the message is creatively and imaginatively crafted, is delivered by trusted members of the community, taps into deeply held beliefs, and involves an engaging story. For example, research has shown that fear appeals alone do not work, particularly when the concept of God is salient, as the receiver may imagine unlimited divine support (Wu and Cutright 2018). Indeed, the use of fear appeals as the main “story” in the battle against COVID has been actively discouraged due to the fact that they overcomplicate an already complex phenomenon (Stolow et al. 2020). Conversely, messages that convey a sense of hope and altruism aid in vaccination acceptance (Wing-Ying and Budenz 2020). We suggest that revision to such Hindu rituals as discussed help create awareness of the problem and the behavioral measures that need to be taken to prevent spread and contagion. Rituals, therefore, may be one platform for delivering such powerful messages. These messages should resonate with the individual or group and have deep emotional and/or spiritual meaning. Religious leaders could work with scientists to create a message that resonates with their members. However, the message must come from trusted religious leaders within the faith. Message creators should also carefully consider their target audience and account for such factors as literacy levels. All too often it is the disadvantaged members of society who are most at risk; therefore, messages that are easy to understand and contain stories, symbols, myths, and characters that convey empathy and sympathy (see, e.g., Escales and Stern 2003) are likely to appeal. In many places, there is a lack of access to the internet, and consequently shared stories and experiences may require a move back to more traditional forms of communication (i.e., through cinema [which is very popular in India], television, and storied advertisements).

One strategy is to carefully craft the message so as to imbue the divine with science (the medicalization of deities) and science with the divine (the sacralization of medics and scientists) so that they are not seen as polar opposites or, indeed, working in opposition. On the contrary, the message should stress that the combined strength of both creates a powerful weapon in the battle against a common enemy. It may further be useful to medicalize the religious rituals to mobilize masses who may rely solely on faith-based practices.

### Create Feelings of Unity and Agency

Staging vivid battles between good and evil, creating anthropomorphic depictions of the coronavirus, and encouraging audience participation are likely to imbue people with a greater sense of unity and confidence. These findings are consistent with Hart’s (1993) description of official actors’ employment of symbolic tools such as rituals during times of crisis. During such times, it is deemed political sagacity to first dramatize the enormity of the issue by ways such as “personifying threats” and “constructing diabolical enemy images” before taking more stringent measures (Hart 1993, p. 42). We recommend consciously revising some elements of popular religious rituals or repurposing them for greater community cohesion and public health. In addition, where feasible, it may be beneficial to recontextualize popular rituals to foster a feeling of unity and community (e.g., the lamp-lighting ritual) as well as well-being and agency (e.g., the fire rituals). As we have shown in this study, the purpose of these revised rituals is not only to help fight the virus but also to give people a sense of control and unity and foster confidence that something is being done.

### Amplify Local Ritual Modifications on a National Scale

The megaphone effect exerted a noticeable influence in our data. Press and media should carefully monitor ordinary consumers who have acquired a large following through their online posts and videos. They and their positive ideas must be further amplified through regional and national media. It will be helpful to have wide media coverage of any megaphone effects, even if they are only visible locally. Our findings echo Scott et al.’s (2020, p. 261) editorial note on the use of social media communication and information sharing as “primary weapons” to inform and illuminate societies about health management during COVID-19.

The theoretical concepts we have developed here (ritual cases, artifacts, and scripts; ritual mobilizers and amplifiers) should apply to other consumer rituals facing crises as well. This applies, for example, to New Orleans Mardi Gras after Hurricane Katrina (Belk et al. 2018; Weinberger and Wallendorf 2012). One thing that we have not covered that Belk et al. (2018) discuss is the emergence of new ritual celebrations in response to overcoming a crisis. Nevertheless, there are still likely to be ritual cases, artifacts, and scripts as well as ritual mobilizers and amplifiers in such commemorative celebrations. The use of these concepts can also be seen, retrospectively, in changes in response to the September 11 World Trade Center attack and collapse (Marcoux 2017). As Marcoux shows, these changes in rituals in response to the crises reverberate and continue to evolve in the years that followed as shown by the changes in the meaning of ritual artifacts. In addition, we can see changes to religious rituals in a small town hit by a devastating tornado (Baker and Baker 2016). In this case, the destroyed church building was gradually restored as the members moved from a tent to a trailer to a new church building. The constructors of the new building paid careful attention to preserving and reconstructing ritual artifacts and scripts, made possible through the actions of ritual mobilizers and amplifiers. These elements continue in the background postcrisis but become visible and salient during the time of crisis and its immediate aftermath. Ritual elements alter as needed through the crisis, and they continue to evolve to meet changing conditions and needs as the crisis becomes largely forgotten, made salient only during times of yearly ritual demarcation (Marcoux 2017).

Our study reinforces the fact that while religious rituals have always been powerful carriers of messages (in many religions), they themselves change with the times (e.g., the Catholic Church’s Latin Mass largely replaced by vernacular Mass). This does not mean that the whole ritual changes; it may be a few elements or just one element. Certainly, there are times when the tradition/ritual “has” to change, because the consequences of not changing may be catastrophic.

## Conclusion

We hope that our observations and analysis will help shed light on several pressing issues. The revamped religious rituals we studied may be temporary and last only until the COVID-19 crisis recedes. However, they provide some important insights about how ritual revision can serve as a way to foster solidarity and engender a sense of perceived agency in expelling threat/evil in a time of catastrophic disease outbreak. This study explores cultural representations in the media and how they allow access to and promote shared interpretations of ritual revision. For this purpose, we gathered data from online news articles, YouTube videos, and YouTube comments. Further studies may be conducted into similar cultural representations on other social media platforms, including Facebook, Twitter, Snapchat, and TikTok. In addition, our findings concerning the outcomes of ritual revisions affecting the general public are limited by our data sources. To acquire a more emic perspective on the issue of ritual revision and its benefits or detriments, in-depth interviews are needed in future research.

Recently, World Health Organization Chief Tedros Adhanom Ghebreyesus warned against a more overwhelming disease outbreak in the future and urged nations to invest in their health infrastructures. We suggest that we can usefully communicate information and develop awareness about diseases and viruses by medicalizing religious discourse and anthropomorphizing the virus. It is important to leverage macro and micro-influencers as well as to utilize the press and media's amplificatory potential. In these ways, the state can capitalize on prevalent megaphone effects and involve different community stakeholders. The revised Hindu religious rituals that this article analyzes can encourage discussion of the ways other practices can be modified and invented during outbreaks for public benefit.

## Notes

1 See https://www.youtube.com/watch?v=nnSaE5qP6Os.
